# Balancing Between Negative Appendectomy and Complicated Appendicitis: A Persisting Reality Under the Rule of the Uncertainty Principle

**DOI:** 10.7759/cureus.81516

**Published:** 2025-03-31

**Authors:** Stylianos Roupakias, Katerina Kambouri, Angelos Al Nimer, Konstantina Bekiaridou, Evangelos Blevrakis, Christos Tsalikidis, Xenophon Sinopidis

**Affiliations:** 1 School of Medicine, University of Patras, Patras, GRC; 2 Pediatric Surgery, University Hospital of Alexandroupolis, Alexandroupolis, GRC; 3 Surgery, University of Gothenburg, Gothenburg, SWE; 4 Pediatric Surgery, University of Crete, Heraklion, GRC; 5 General Surgery, University Hospital of Alexandroupolis, Alexandroupolis, GRC

**Keywords:** children, complicated appendicitis, diagnostic methods, diagnostic uncertainty, negative appendectomy

## Abstract

Uncertainty is inherent in medical practice. False-negative decisions can delay treatments and result in adverse outcomes in children with acute appendicitis (AA). On the other hand, false-positive surgery decisions lead to unnecessary appendectomies. Impressive technological advancements, such as magnetic resonance imaging and laparoscopy, have reduced but failed to eliminate the occurrence of erroneous decisions. Furthermore, there seems to be a fundamental limit to further reduction, especially in eliminating the rates of negative appendectomy or, oppositely, complicated appendicitis. What does this mean for the pediatric surgeon? Will we ever be able to eliminate our mistakes? This systematic review emphasizes the importance of understanding the potential abilities and limitations of different diagnostic options, as well as the impact of decisions in the face of uncertainty.

## Introduction and background

The fundamental principle of uncertainty is not a limitation of science’s ability to measure, but rather an inherent property of the physical world. On a human scale, these errors may seem trivial but cannot be ignored [1]. Medical practice is inherently full of uncertainties [2], which affect the relationship between a patient's well-being and diagnostic knowledge regarding the underlying disease [3]. At times, confusion arises in distinguishing between medical and personal knowledge [4]. The basic process of prompt and appropriate diagnosis is often challenging due to both understood and misunderstood medical factors and variables, which introduce uncertainties [1]. These factors include disease presentation variability, symptom overlap from different conditions, limitations of available diagnostic tests, constraints on medical technology and knowledge, and the physician’s inexperience in interpretation. The uncertainty principle in medicine underscores the importance of informed decision-making, considering the odds and risks associated with various diagnoses and treatments. This process may involve ongoing monitoring and reassessment of the patient's condition, as well as flexibility to adjust treatment plans as new information or circumstances arise. In addition, it highlights the need for the improvement of diagnostic tools to reduce uncertainty and enhance patient outcomes.

Medical uncertainties can cause anxiety and stress for healthcare professionals, as they may lead to diagnostic errors and increase the risk of adverse outcomes. Healthcare professionals may also feel uncertain about the optimal timing for action and often rely on their own clinical judgment and experience to make decisions and interventions. While future technologies and precision medicine, including advanced information systems and artificial intelligence, may reduce uncertainty, they could paradoxically introduce new, unforeseen types of uncertainty [5]. Even in the era of the fourth industrial revolution, uncertainty in medicine may persist or even grow [5], rendering medicine both a science of continuous uncertainty and an art of probability.

In pediatric surgical clinical practice, peritonitis caused by the delayed diagnosis of acute appendicitis (AA) remains a persistent issue. On the contrary, unnecessary appendectomies of a normal appendix, often referred to as negative appendectomies, continue to occur. However, in most cases, these are simply unnecessary procedures rather than true-negative appendectomies. In this review, we aim to explore this contradictory reality through the lens of the universal principle of uncertainty.

## Review

In medical practice, diagnostic uncertainty, defined as the subjective inability to provide timely and accurate medical management to patients, is an inherently dynamic state [6]. It is particularly evident in emergency situations [3]. Uncertainty is a common challenge in diagnosing appendicitis, especially in children. Younger children have a lower incidence of appendicitis, but their nonspecific symptoms, less reliable history, and delayed diagnosis often lead to perforation and complicated disease progression [7-12]. Clinical examination of children requires special skills and patience, as they tend to be uncooperative, more irritable to touch, and unable to localize pain [7]. To help diagnose appendicitis, physicians often use a combination of physical examination and diagnostic tests, such as blood tests and imaging studies. A watchful waiting approach may be appropriate when the diagnosis remains uncertain, particularly in cases where the likelihood of appendicitis is borderline or when other comorbidities are present. In these cases, appendectomy may be recommended as the definitive diagnostic and treatment approach.

Physicians need to understand the potential risks and benefits of different treatment options and decisions in the context of uncertainty. Diagnostic uncertainty in medical management can lead to both overdiagnosis and overtreatment of presumed appendicitis, as well as underdiagnosis, resulting in delayed appropriate treatment [13,14]. Perforated appendicitis due to physician misdiagnosis is considered underdiagnosis or false-negative decision errors, while negative appendectomies are considered overdiagnosis or false-positive decision errors [14]. False-negative decisions and the resulting delay in appendectomy are a leading cause of expensive malpractice claims in emergency medicine in the United States [15]. On the other hand, false-positive decisions and the subsequent often unnecessary negative appendectomy contribute to higher healthcare costs and an avoidable procedure for pediatric patients.

Acute appendicitis in children

AA in children is one of the most common causes of abdominal pain requiring urgent surgery [16]. Its atypical and/or vague presentation often leads to a wide range of differential diagnoses, increasing the risk of delayed diagnosis, perforation, and consequently higher morbidity, prolonged hospitalization, and even mortality in young children [17]. Gastroenteritis, mesenteric adenitis, constipation, ovarian cysts or torsion, and other conditions are the most common causes of acute abdominal pain in children that mimic appendicitis [7].

Complicated appendicitis is defined as inflammation accompanied by gangrene, perforation, periappendicular phlegmon, perityphlic abscess, or free purulent fluid [18]. Children with complicated appendicitis are at increased risk for many complications such as wound infection, postoperative abscess, sepsis, wound dehiscence, prolonged ileus, and delayed bowel obstruction [19]. The reintervention rate is higher in complicated appendicitis cases [20,21]. The incidence of pediatric perforated appendicitis ranges between 20% and 74%, with higher rates in children aged two to five years (69%-93%) and up to 100% for infants [22,23]. A study of 55,591 appendectomies in children found a 21% perforation rate [24]. Perforation and periappendiceal abscess rarely occur within the first 12-24 hours of symptoms onset. They are more likely to occur after 36-48 hours and most commonly after 48-72 hours [23,25-29]. The perforation rate is a poor prognostic factor, and it doubles when surgical management is delayed beyond 24 hours [19,30]. A delay of up to 12 hours in children, with antibiotics administered, does not significantly affect the morbidity of uncomplicated appendicitis [18]. Perforated appendicitis increased the rate of postoperative wound infections from under 5% to 20% [31]. With an average perforation rate of 20%, the average abscess rate is about 7% [19]. Surgeons who delay surgery to clarify the diagnosis may miss the opportunity to remove the appendix before perforation occurs. Appendectomy delay is associated with a significantly increased risk of surgical site infection in patients with nonperforated appendicitis [32].

To avoid the morbidity associated with undiagnosed perforated appendicitis in the general population, negative appendectomy rates of up to 25% have been justified. In the pediatric population, the acceptable rate is even higher [33]. However, reported rates of negative appendectomy in pediatric literature have continued to vary widely, ranging from 1% to 40% [34-43]. In large pediatric populations with appendectomies, the reported rate of negative appendectomy ranges from 4.3% to 6.7% [34,44,45]. As with any surgery, complications such as infection, bleeding, and iatrogenic injury may occur, rendering operative exploration for appendicitis not without risk [33]. Complication rates are not lower when removing a normal appendix compared to an inflamed one, with the same severity and reoperation rates [46]. A national review of patients who underwent negative appendectomy showed increased length of stay and complication rates compared to those with uncomplicated appendicitis [41]. Furthermore, readmissions for persistent abdominal pain are remarkable after the removal of a normal appendix [33].

The vermiform appendix is ​​now recognized as an important component of the intestinal immune system, playing a vital role rather than being an unnecessary evolutionary remnant [47,48]. Research has shown that the appendix contains a significant amount of lymphoid tissue, which plays a crucial role in the body's immune response [47,48]. The appendix serves as a reservoir for beneficial bacteria that live in the gut, helping repopulate the intestinal flora after an infection or antibiotic treatment [48]. Furthermore, the appendix may play a role in the development of the immune system during early childhood. It has been suggested that the appendix helps train the immune system to recognize and tolerate harmless antigens and also helps stimulate the production of antibodies [48]. An early appendectomy may be associated with an increased risk of several diseases, as it could significantly alter the gut microbiome and reduce intestinal immune reactivity [49-52]. Therefore, any intervention such as appendectomy could have a long-term impact on any developing organism with a long life expectancy, let alone in children.

Thus, if complications such as perforation, periappendiceal abscess formation, and sepsis can be associated with false-negative physician decisions, then unnecessary appendectomies can be associated with false-positive physician decisions [19]. As early as 1984, Berry and Malt interestingly found a primary linear correlation between higher physician diagnostic accuracy and higher perforation rate [53].

False-negative decisions

False-negative decisions in the diagnosis of AA occur when a child with AA is incorrectly diagnosed as not having it. The diagnosis of AA is missed in 3.8%-15% of children [12,54,55]. Underdiagnosis of AA can occur for several reasons, including atypical symptoms, delay in seeking care or medical attention, physician’s inadequate diagnostic training and experience, incomplete examination or lack of appropriate imaging tests, and misdiagnosis of other conditions, all of which contribute to delayed surgical intervention. One study found that delays in hospital admissions by emergency physicians are a more significant cause of complicated appendicitis and postoperative complications than patient delays in seeking care and surgeon delays in performing the operation [15]. False-negative assessments occur when the physician initially examining the patient fails to make the diagnosis promptly, leading to delays in surgical intervention [19]. Failure to refer the patient for a surgical evaluation increases the risk of perforation and subsequent complications [19].

The diagnosis of AA is potentially missed in 4.4% of children during the initial emergency department visit [56] and is only made after two or more visits in 15% of younger patients [12]. Missed diagnoses are more common in children younger than five years, girls, those with comorbidities, and those experiencing abdominal pain accompanied by constipation [56]. A surgeon’s low confidence refers to a situation where a surgeon lacks confidence in their abilities or decision-making skills and opts for conservative management of acute abdominal pain, delaying surgical intervention. This reluctance can be caused by several factors, including inexperience, fear of making mistakes, or lack of support from colleagues or superiors. Low confidence can negatively affect patient outcomes, leading to suboptimal decisions and delayed procedures. The rate of false-negative decisions ranges from 10.6% to 27.8%, with more than 20 hours of delay in surgery from 1.5% to 18.9% [19]. Overall, timely diagnosis and prompt surgical intervention are critical in preventing complications of appendicitis in children, though some cases of AA will inevitably be missed.

False-positive decisions

False-positive decisions in the diagnosis of AA occur when children are incorrectly diagnosed with the condition. Such misdiagnoses can lead to unnecessary surgical interventions, increasing medical costs and exposing patients to potential risks of intraoperative or postoperative complications, as well as prolonged hospital stays or readmissions. Overdiagnosis and negative appendectomy reveal a possible overreliance on clinical symptoms alone and the reluctance of the surgeon to risk missing a potentially more serious condition, such as complicated appendicitis.

Surgeon overconfidence refers to the tendency of some surgeons to opt for more aggressive management of acute abdominal pain, believing they can easily perform surgery without any hesitation, delay, or concern for potential complications. This overconfidence can be influenced by several factors, including experience, personality, and prior success rates. Surgeons with extensive experience and a history of successful surgeries may be more prone to overconfidence, as they may feel that they have encountered every scenario and know what to expect. In some cases, an experienced clinician's judgment might justify surgical intervention, even in the absence of pathologic inflammatory markers [57].

When the consequences of an incorrect treatment decision are considered, the threshold for initiating surgical treatment is likely to be lowered. Therefore, alongside the increasing trend of early diagnosis of AA, even in cases with equivocal imaging findings, there is also a trend toward overtreatment in the pediatric population [13]. Physicians, uncomfortable with uncertainty, often instinctively treat what they perceive as a major problem [1]. However, it is important to remember that a physician's convenience may not always align with the patient's best interest [1]. Negative appendectomy rates in children have decreased over time, likely due to advancements in imaging techniques.

Eliminating the appendicitis diagnostic uncertainty errors

Physicians who are intolerant to uncertainties tend to prescribe excessive amounts of diagnostic tests [58]. To minimize the risk of false-positive and false-negative decisions in managing children with acute abdominal pain, physicians may use a combination of diagnostic tools and physical examination to increase the accuracy of diagnosing AA. They may use clinical scoring systems, such as the modified Alvarado score for children or the Pediatric Appendicitis Score, to help identify patients who are more likely to have AA and require surgery [59,60]. Both scoring systems can be of assistance in setting the diagnosis of AA, but none has adequate predictive values in assessing AA, and none can be used as an exclusive standard in setting the diagnosis of AA in children [61]. It seems evident that the opinion of an expert surgeon can never be replaced by a scoring system, and that the final decision whether to operate or not must rest on his criteria [61]. In addition, advanced imaging studies such as ultrasound (US), computed tomography (CT) scans, or magnetic resonance imaging (MRI) can help confirm the presence of AA and rule out other conditions with similar symptoms. The use of US and CT imaging in the pediatric population has increasingly played a role in the prompt and accurate diagnosis of AA over the last decade. Not only did they improve the rupture rates but also reduced the negative appendectomy rates [16,25].

Although a decrease in the frequency of negative appendectomies due to diagnostic uncertainty has been observed, it nevertheless remains notable [19]. According to a study on a huge sample of children with appendectomies, the rate of negative appendectomy has decreased from 21.4% in 1987 to 6.5% in 2009 [24]. However, many cases of AA remain undiagnosed [19]. A current pediatric study showed an improvement in the rate of undiagnosed appendicitis to 4.8% among 816 children at their first medical visits [54]. Sometimes, just waiting and seemingly doing nothing is an alternative therapeutic modality, which does not necessarily mean doing nothing [54]. Because of the fear of missing a treatable disease, we have forgotten the precept of masterly inactivity [62]. In any child with acute abdominal pain, especially in the absence of convincing clinical examination findings, advanced imaging methods, and vague imaging results, close follow-up with sequential clinical abdominal examinations (preferably by the same surgeon) is mandatory. Definite diagnosis should be postponed. Intensive observation with reexaminations every eight hours is safer and more effective than the approach of once-per-day reassessment in a hospital [63]. Although less frequently, the interpretation of imaging tests can be subjective and may also lead to false-positive or false-negative appendicitis diagnoses.

In a minority of children, the final diagnosis will still be uncertain. No pathological imaging diagnosis can provide indisputable safety against “overdiagnosis” by well-intentioned radiologists or, under these circumstances, equally well-intentioned pediatric surgeons [13]. However, at a certain point, making a therapeutic decision is imperative, and no further research may provide more certainty [63]. Concurrently accurate and timely diagnosis cannot be precise and is always accompanied by relative uncertainty. This uncertain medical situation cannot be time-consuming. Surgeons who can master their overconfidence and insecurity, either by meticulous and immediate intervention or with masterly inactivity, are best equipped to vanquish uncertainty and serve children well [64].

Application of the uncertainty principle in pediatric appendicitis

The application of the uncertainty principle in pediatric AA describes a trade-off between the rates of negative appendectomy and the incidence of complicated appendicitis. It is a fact that the frequency of both negative appendectomies and complicated appendicitis due to diagnostic uncertainty has been significantly reduced, but it can neither be ignored nor completely eliminated. Is there truly an inherent uncertainty in nature that we cannot reduce below a certain threshold? Is there some foundational upper limit to accuracy by which the coupled variables of complicated appendicitis and negative appendectomy cannot be further simultaneously reduced? Attempting to eliminate negative appendectomies leads to an increase in complicated appendicitis, while focusing on complicated appendicitis results in a higher rate of negative appendectomies. A critical component of clinical decision-making is a thinking process of diagnostic reasoning, which refers to the cognitive processes that healthcare professionals use during medical practice. It forms the core of professional autonomy [65]. The uncertainty principle indicates by signs an amount of complete pediatric surgeon’s reasoning autonomy and responsibility of choice.

Gain the limit by imaging

What exactly is this threshold, and what factors does it depend on? Below this threshold, an increase in the value of one error leads to a decrease in the value of the other. Once AA is suspected, no single history, physical examination, laboratory finding, or scoring system can eliminate the necessity for imaging studies [66]. Appendectomy should not be undertaken without imaging to confirm the clinical suspicion, but there will never be a single globally accepted strategy for the evaluation of possible appendicitis in children [67,68]. No imaging modality possesses 100% sensitivity and specificity simultaneously; thus, it can never definitively exclude or confirm the disease in every case.

Computed Tomography

CT is the most accurate imaging modality for a suspected AA [69]. There is a significant inverse relationship between the annual increase in the rate of CT use and the annual decrease in the overall rates of false-positive diagnosis of appendicitis and perforated appendicitis [70]. A meta-analysis revealed a significantly higher negative appendectomy rate in the pre-CT era compared to the post-CT era (21.5% vs. 10%), while the incidence of appendiceal perforation remained statistically unchanged [71]. In the United States, CT is routinely performed in 20%-95% of patients, presumably contributing to the <5% rate of negative appendectomies [72]. Regarding the pediatric population, a study by Mahajan et al. found that 1.3% of children with potentially undiagnosed appendicitis underwent a CT scan at the initial visit [56]. The long-term effects of ionizing radiation from CT scans in the pediatric population must be taken into consideration when weighing the risk of future malignancy against the potential consequences of missing an appendicitis diagnosis [73]. For children younger than 15 years, the estimated risk of death from radiation-induced malignancy ranges from 0.07% to 0.10% [74]. The risk of lifetime mortality from cancer due to abdominal CT in a one-year-old child is 0.18% (one in 550) [74]. In a recent population-based case-control study, participants exposed to four or more CT scans (especially before the age of six) had an increased incidence of leukemia, intracranial tumors, and lymphomas in childhood and early adulthood [75]. Exposure to potentially hazardous contrast material is another disadvantage [7]. The sensitivity, specificity, and accuracy of non-enhanced CT are 97%, 100%, and 98%, respectively [76]. There is insufficient evidence to support the routine use of CT scans in children [38], and they should be reserved as a last option [7].

Ultrasound Imaging

Growing concerns over medical radiation have shifted the preferred diagnostic method from CT to ultrasonography, especially in children [13,77]. In cases of diagnostic uncertainty, serial US examination can reduce the need for CT scans without increasing undue risk to the patient [78]. Since US findings are more subjective and dependent on operator experience, they are associated with a significantly higher rate of indeterminate and false-positive results compared to CT [13]. In a study, 11.2% of children with US-positive appendicitis had CT-negative appendicitis, and 31.9% of children with US-negative appendicitis had CT-positive appendicitis [79]. A US-negative finding may not suffice to rule out appendicitis [66,80]. Furthermore, the appendix is often difficult to visualize in children with retrocecal appendices [56]. CT is performed more often in obese patients, because the ultrasonographic signs of appendicitis are hard to evaluate in this patient group [81]. In a prospective multicenter study evaluating US performance in children, 51% of cases yielded indeterminate results [82]. Another retrospective review of all sonograms for AA in children showed that the appendix was identified only in 246/1009 cases (24.4%), 35 being false-positives and 54 false-negatives [83]. In 223 appendectomies performed on pediatrics, the rate of negative appendectomy was 3.6% and the rate of complicated appendicitis was 5.8%, with US as the only imaging modality [84]. Nowadays, US sensitivity in children is estimated at about 83%-99% and its specificity about 87-100% [77]. A serial (initial and interval) US diagnostic pathway in suspected appendicitis has significantly higher diagnostic accuracy (97% sensitivity, 91% specificity) than that of the initial US, and results in fewer CT scans [85]. In the hands of experienced practitioners, the accuracy of US approaches that of CT or MRI scans [86]. The use of US in children is accurate and safe in terms of perforation rates, emergency department revisits, and negative appendectomy rates [87].

Ultrasound Combined With Computed Tomography

Under an imaging cooperative protocol, known as Poortman’s model [88], clear US signs cannot definitively “rule out” AA; therefore, a CT scan is employed [69,88]. After the implementation of the US/CT imaging protocol in children with suspected appendicitis, the perforation rate decreased from 35.4% to 15.5%, and the negative appendectomy rate decreased from 14.7% to 4.1% [89]. In a population of 385 boys (8-14 years) who achieved a negative appendectomy rate of only 1%, 30% of them underwent CT, 51% had only US, 12% had both US and CT, and 7% had no imaging in their evaluation [90].

Magnetic Resonance Imaging

To mitigate the hazards associated with CT, MRI is gaining popularity as an alternative imaging modality [7]. A recent study showed high MRI accuracy in children with 96% sensitivity and specificity [91]. In a study of 77 performed MRI, one patient had false-positive result and two had false-negative results [92]. Although MRI shows promise, its use is limited due to availability and cost considerations [51]. Children under the age of five often require sedation or general anesthesia for MRI [18].

Ultrasound and Magnetic Resonance Imaging

A staged, radiation-free US/MRI imaging algorithm for diagnosing AA appears to be effective, beneficial, and preferable in children [93,94]. In a cohort study by Dibble et al., the rate of negative appendectomy was 0.2% (four of 1982 patients), much lower than the previously reported overall institutional rate of <2% [93]. A US/MRI algorithm presented 98% sensitivity and 97% specificity, with 45 false-positive and seven false-negative results [92].

Reviewing systematically the literature in the last decade

**Figure 1:**
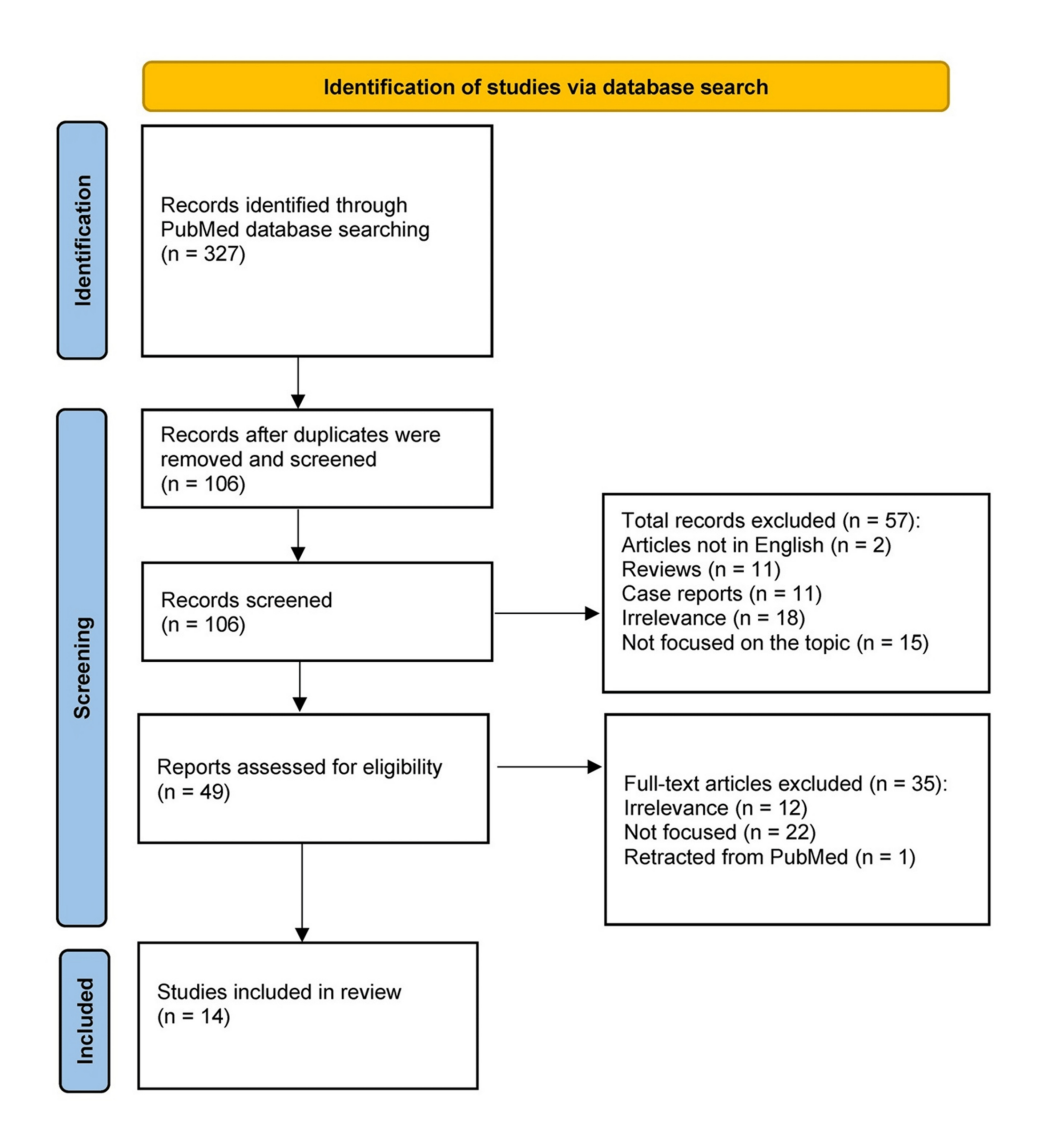
Flow diagram of literature search and article selection process according to the PRISMA guidelines. PRISMA: Preferred Reporting Items for Systematic Reviews and Meta-Analyses.

We performed a short systematic review to identify the articles that reflected the most updated trends on the topic of the diagnostic accuracy of US, CT, and MRI, and their association with clinical parameters. We searched the PubMed database and used the Preferred Reporting Items for Systematic Reviews and Meta-Analyses (PRISMA) statement flow diagram to identify the most appropriate outcomes [95]. Keyword combinations of the terms "appendicitis", "missed diagnosis", "false diagnosis", "false negative diagnosis", "false positive diagnosis", "ultrasound", "computed tomography", "MRI", and "children" were used. The research period included all published original articles from January 2014 to December 2024. Studies on adults, written in a language other than English, and of irrelevant content to the topic of diagnostic accuracy in appendicitis in children were excluded. Including a systematic review in this paragraph on the diagnostic adequacy of modern imaging facilities, within the broader context of multiple parameters, a short study period, and reliance on a single database (PubMed), could introduce research bias. Still, we consider its outcomes fairly representative, as our aim was not to exhaustively review the imaging factor but to holistically assess the clinician’s challenge when diagnosing possible appendicitis in a child. The flow diagram of this investigation is shown in Figure 1. The main characteristics and outcomes of the 14 included articles are summarized in Table 1 [85,92,96-107].

**Table 1:** Original articles of the last decade, which represent the contemporary diagnostic trends, combining radiological examinations and clinical motives. US: ultrasound, CT: computed tomography, MRI: magnetic resonance imaging, PPV: positive predictive value, NPV: negative predictive value, SD: standard deviation, FP: false-positive tests, FN: false-negative tests, PAS: pediatric appendicitis score.

Author	Year	Country	Study design	Number of patients	Age (mean)	US outcomes	CT outcomes	MRI outcomes	Combined outcomes	Important article topics
Schuh et al. [85]	2015	Canada	Prospective cohort study	294 children had an initial US study (boys, 48%)	10.4 years	Serial US pathway showed 93% diagnostic accuracy, with a sensitivity of 97%, specificity of 91%, PPV of 86%, and NPV of 98%	The rate of CT in the study was negligible	N/A	N/A	A pathway of US (initial and interval), combined with PAS was implemented to obtain high-accuracy diagnostic levels
Dibble et al. [92]	2017	USA	Retrospective study	2180 children: 1023 (46.9%) boys and 1157 girls	Boys: 10.6 years, age range 1.2-18 years Girls: 12.7 years, age range 0.6-18 years	Appendicitis diagnosis presented a sensitivity of 98.2%, specificity of 97.1%, and NPV of 99.5%	N/A	Unenhanced MRI presented a sensitivity of 87.5% and specificity of 97.1%	The combination of US and MRI was proved highly effective. The false-negative rate of the algorithm was 0.4%	A staged algorithm including US and non-enhanced MRI (for equivocal US results) was tested for the diagnosis of appendicitis in children
Unsdorfer et al. [96]	2021	USA	Retrospective and interventional study	1075 children were included in the post-implementation study: 604 (56.2%) girls and 471 (43.8%) boys	11 years pre- and 10 years post-implementation	Sensitivity from 94.8% to 91.3%, specificity from 96.3% to 98.1%, PPV from 86.5% to 87.9%, and PNV 98.7% without change. Overall accuracy from 0.960 to 0.972 and error rate from 0.040 to 0.028	N/A	N/A	N/A	The researchers, after having retrospectively studied US outcomes, performed an educational intervention and reevaluation
Kim et al. [97]	2020	South Korea	Retrospective study	292 children underwent US, CT, or both for acute appendicitis	8.9 years	18 patients with true-negative results, 23 patients with false-negative results	After US was performed, 41 patients underwent confirmatory CT	N/A	CT scans could have reduced the false-negative group by 100%	Vomiting, high WBC, and high CRP are suggestive factors of the false-negative group with US
Heye et al. [98]	2020	USA	Retrospective study	350 patients were submitted in unenhanced and non-sedated MRI: 206 (58.9%) girls and 144 (41.1%) boys	12 (age range, 3-18) years	N/A	N/A	65 positive for appendicitis with a false-positive rate of 6/65 (9.2%), 265 negative for appendicitis with a false-negative rate of 2/265 (0.8%), 29 (8.3%) equivocal studies. Method sensitivity 96.7%, specificity 97.7%	Previous US and CT were considered non-diagnostic.	The overall diagnostic accuracy is 97.5%. The method showed characteristics of high diagnostic accuracy. PPV 90.8%, NPV 99.2%
Davis et al. [99]	2020	USA	Retrospective study	88 patients: 30 (43.1%) boys and 48 (65.9%) girls	10.1 years	The authors considered a non-visualized appendix as normal, with a sensitivity of 42% and a specificity of 97%.	N/A	N/A	N/A	The overall accuracy values were lower than those in children-oriented tertiary centers. PPV 80%, NPV 86%, FP 20%, FN 14%
Corson-Knowles and Russell [100]	2018	USA	Prospective observational study	105 patients (24 children)	23.9 ± 13.1 years	Faculty members presented a sensitivity of 50%, fellows 75%, and residents 31%. The total sensitivity was 43%. Faculty members presented a specificity of 100%, fellows 100%, and residents 96%. The total specificity was 98%	N/A	N/A	N/A	Emergency medicine participants received a short US training in appendicitis identification
Binkovitz et al. [101]	2015	USA	Retrospective study	790 children with US: 452 (57%) girls and 338 (43%) boys	10.4 years	Definitive studies presented 94.8% sensitivity, 96.3% specificity, 96.0% accuracy, PPV 86.5%, and NPV 98.6%. Total outcomes presented a sensitivity of 74.7%, specificity of 69.3%, and accuracy of 74.7%	N/A	N/A	N/A	The US reports were categorized into definitive and indeterminate diagnoses and were evaluated on their accuracy. Accuracy was influenced by appendicitis severity, age, gender, BMI, symptom duration, or call status
Cohen et al. [102]	2015	USA	Retrospective study	1383 US performed in 1260 patients for appendicitis evaluation	Age range: 0-18 years	202 (14.53%) positive studies, 83 (6%) suspicious, 17 (1.23%) low suspicion, 152 (10.99%) negative, 54 (3.90%) showed other pathology, and 876 (63.34%) were non-diagnostic for acute appendicitis	N/A	N/A	N/A	The higher NPV non-diagnostic for appendicitis and non-visualized appendices were associated with WBC values under 7.5 x 10^9^/L (97.12% and 98.86%, respectively)
Bachur et al. [103]	2015	USA	Retrospective study	728 children with US and PAS: 317 (44%) boys	11.7 years	US findings were positive (22%), equivocal (22%), and negative (56%). US sensitivity was 86%, specificity 74%, NPV 93%, and PPV 57% in equivocal positive results. Contrarily, in equivocal negative results, sensitivity was 68%, specificity 98%, NPV 88%, and PPV 92%	N/A	N/A	N/A	The association of US with clinical presentation showed that US results combined with low clinical scores need observation and/or further assessment
Tunc et al. [104]	2021	USA	Retrospective study	1874 evaluated participants (girls, 53%)	11 years	The rate of negative appendectomy was statistically 1.6% vs. 2.4% after the diagnostic pathway implementation	N/A	N/A	The initial rate of CT rate decreased from 31% to 17%, with an associated increase of US from 83% to 90% after the diagnostic pathway implementation	Pediatric patients were evaluated for possible appendicitis with and without the use of a diagnostic pathway including PAS and US
Aydin et al. [105]	2018	Turkey	Retrospective study	212 children with US (119 with appendicitis)	11.5 ± 3.6 years	US was positive in 88.2% of the appendicitis group and 33.3% in patients without appendicitis (US sensitivity 88.2%, specificity 66.7%, NPV 81%, PPV 77%). Combined with PAS, US sensitivity was 91.1%, and specificity 71.1%	N/A	N/A	N/A	The association between PAS and US was studied in terms of its diagnostic accuracy. When discordance exists between US and clinical assessment, serial examinations, observation, or further imaging are warranted
Chien et al. [106]	2016	USA	Retrospective study	496 evaluations prior to and 418 after the implementation of a clinical and US pathway were studied	12.7 vs. 13.3 years before and after the pathway	US sensitivity was 96.3% and specificity 99%. US-positive rate improved from 33.3% to 54.0%	CT rates fell from 91.7% prior to the implementation of the pathway to 25.1% after	N/A	N/A	An associated diagnostic pathway with clinical parameters and US showed a decline in CT use
Blitman et al. [107]	2015	USA, Pakistan	Retrospective study	522 children (231 boys, 291 girls)	13.04 years	US sensitivity was 67.6% and specificity 98.1%. The NPV of inconclusive focused appendicitis US and low Alvarado scores was 99.6%, while in cases of higher Alvarado scores decreased to 89.7%	N/A	N/A	Though CT was not designated for the study, the rate of CT (17.8%) in the low Alvarado score would have been avoided. The total reduction in CT would be 42.8%	US and Alvarado clinical score were used together to evaluate combined diagnostic accuracy

The first outcome of this investigation was that most studies were retrospective, with only two [85,100] of a prospective cohort design. There was a lack of double-blinded studies. Ultrasonography tends to prevail as the most popular investigation among CT and MRI, with high diagnostic accuracy [85,92,96,97,99-107]. Studies with lower sensitivities and predictive values were from a community hospital [99]. All others were from tertiary university hospitals. In an observational prospective study, emergency medicine clinicians demonstrated high specificity in diagnosing AA, after a brief 20-minute hands-on education with a healthy subject. This training improved clinical US performance in an appropriate clinical context, showing that ultrasonography can achieve optimal diagnostic accuracy, even when performed by clinicians, after proper and ongoing education by radiologists [100]. In studies involving CT, there was a clear tendency to reduce its use, reserving it only for difficult diagnostic cases [97,104,107], though MRI, with its novel aspects, such as non-enhanced types, tends to take the place of CT for these cases [92,98]. Although ultrasonography is evolving and gaining traction, it is clear that the highest diagnostic outcomes are achieved only when it is combined with clinical characteristics, either individually or through clinical pediatric scores such as the PAS or Alvarado score. As a result, modern algorithms aim to incorporate both US and clinical characteristics [85,97,101-107]. The use of serial US in these US-based, clinical-oriented algorithms appears to enhance diagnostic accuracy [85].

Diagnostic laparoscopy

Can the eyes of a surgeon surpass the radiologist’s assessments? About 68% of negative appendectomies could initially be diagnosed intraoperatively as inflamed appendicitis and later prove to be non-inflamed on histological examination [108]. On the other hand, in 18%-29% of macroscopically normal appendix cases, histological study reveals appendicitis or other appendiceal pathology (neoplasia, endometriosis, parasites, and appendicolith) [18]. About 10% of histopathologic perforated appendicitis cases could not be detected during surgery [109]. Does laparoscopy decrease the rate of unnecessary removal of normal appendices? Intraoperative laparoscopic assessment of the appendix can be difficult [96]. In more than half of the microscopically healthy appendices, the surgeon was convinced of an appendicitis diagnosis during surgery [110]. In a study of 200 consecutive laparoscopic appendectomies, 7.2% of macroscopically inflamed appendices were found to be microscopically normal, while 25% of macroscopically normal appendices were found to be microscopically inflamed [111]. The inclusion of laparoscopy as a diagnostic method is primarily supported by surgeons who believe that a negative appendectomy is an unnecessary procedure and would avoid excising an apparently normal appendix. Conversely, those who take the opposite view consider appendectomy routine, regardless of the appendix's appearance. However, laparoscopy in a broader context may be considered diagnostic in cases where the pathology mimics appendicitis, such as omental torsion or other causes of abdominal pain of different origins [112].

Histological diagnosis of acute appendicitis

Large database studies often only report the discharge diagnosis or intraoperative appearance of the appendix, neglecting histological analysis [108]. Additionally, there is controversy over the diagnostic criteria required for the histological diagnosis of AA, and this contributed to variations in reported negative appendectomy rates. Some pathologists define AA as transmural inflammation (neutrophilic infiltration of the muscularis propria), while others suggest that mucosal inflammation in correlation with the patient’s presenting symptoms must be considered as early appendicitis [113,114]. Inflammation of the mucosa of the appendix is often assigned to a differential diagnosis of enteritis or inflammatory bowel disease [113,114]. Pinworms, granuloma, or malignancy can occasionally cause transmural inflammation of the appendix owing to obstruction [33]. The American Association for the Surgery of Trauma developed an appendicitis grading system, based upon specific clinical, radiologic, operative, and pathologic criteria [115]. This grading system focuses on adult pathology but is also applicable to children. It describes pathologically the acutely inflamed intact appendix with the presence of neutrophils at the base of the crypts, submucosa, and occasionally in the muscular wall [116].

Concerns

Contrary to expectations, some authors conclude that the frequency of appendicitis misdiagnosis resulting in unnecessary appendectomy has not changed with the introduction of CT, ultrasonography, and laparoscopy, nor has the frequency of perforation or patient outcomes improved [117,118]. Is it probable that the final decision to overcome the uncertainty will continue to depend significantly on the opinion of an expert pediatric surgeon?

Few studies have shown 0% negative appendectomy or missed CT appendicitis rates, but not perforated appendicitis rates [119-121]. However, the number or characteristics of patients in their study populations was limited. Studies with larger populations and no exclusion criteria obviously reveal increased negative appendectomy rates.

Researchers should interpret their results from the perspective of previous studies and the working hypotheses. Their findings and their implications should be discussed in the broadest context possible. Future research directions may also emerge and should be taken into consideration.

## Conclusions

How can a pediatric surgeon be absolutely certain? Despite advancements in imaging and surgical techniques, negative appendectomy and complicated appendicitis rates cannot be reduced to zero, especially not simultaneously. A systematic institutional approach to pediatric AA should improve outcomes and reduce rates of appendiceal perforations and negative appendectomies, considering the potential long-term risks of appendectomy in children. Addressing this uncertainty requires balancing the risk of removing normal appendices too often versus delaying treatment in cases of perforation.

Multidisciplinary collaboration, evidence-based protocols, and ongoing research into diagnostic advancements are essential to refining this balance and optimizing patient outcomes. Utilizing validated clinical scoring systems, enhancing imaging techniques, and incorporating emerging biomarkers can enhance diagnostic accuracy and guide management decisions. Ultimately, optimizing the management of pediatric AA requires a dynamic, data-driven approach that adapts to the inherent uncertainty of medical practice.
